# Ethnic differences in human papillomavirus awareness and vaccine acceptability

**DOI:** 10.1136/jech.2008.085886

**Published:** 2009-09-17

**Authors:** L A V Marlow, J Wardle, A S Forster, J Waller

**Affiliations:** Health Behaviour Research Centre, Department of Epidemiology and Public Health, UCL, London, UK

## Abstract

**Background:**

Studies of human papillomavirus (HPV) awareness and HPV vaccine acceptability have included few non-white participants, making it difficult to explore ethnic differences. This study assessed HPV awareness and HPV vaccine acceptability in a sample of women representing the major UK ethnic minority groups.

**Methods:**

A cross-sectional study design was used to assess awareness of HPV and acceptability of HPV vaccination. Participants were recruited using quota sampling to ensure adequate representation of ethnic minority women: Indian, Pakistani, Bangladeshi, Caribbean, African and Chinese women (n = 750). A comparison sample of white British women (n = 200) was also recruited.

**Results:**

Awareness of HPV was lower among ethnic minority women than among white women (6–18% vs 39% in white women), and this was not explained by generational status or language spoken at home. In a subsample who were mothers (n = 601), ethnicity and religion were strongly associated with acceptability of HPV vaccination. Acceptability was highest among white mothers (63%) and lowest among South Asians (11–25%). Those from non-Christian religions were also less accepting of the vaccine (17–34%). The most common barriers to giving HPV vaccination were a need for more information, sex-related concerns and concern about side-effects. South Asian women were the most likely to cite sex-related concerns, and were also least likely to believe the vaccine would offer their daughters protection.

**Conclusion:**

These findings suggest some cultural barriers that could be addressed in tailored information aimed at ethnic minority groups. They also highlight the importance of recording ethnicity as part of HPV vaccine uptake data.

The introduction of human papillomavirus (HPV) vaccination aims to reduce the incidence of cervical cancer. The three-dose vaccine is highly effective, providing protection against HPV types 16 and 18, which are responsible for approximately 70% of cervical cancers.^1^ The UK programme offers vaccination to girls aged 12–13 years. It is expected to cost up to £100 m a year and, to maximise its benefits, coverage must be high.^2^ This depends on the vaccine being acceptable to parents.

In the UK, two large studies have explored attitudes to HPV vaccination.^3^ ^4^ ^5^ The main barriers have been concerns about vaccine side-effects and the sexually transmitted nature of HPV infection, which provokes worry that the vaccine will promote sexual activity in adolescents. Similar barriers have been identified around the world.^6^ ^7^ ^8^ ^9^ ^10^ ^11^ In addition, work has shown very low awareness of HPV as a risk factor for cervical cancer.^12^ ^13^

To limit inequalities and support informed choice, it is important to understand levels of HPV awareness and HPV vaccine acceptability across all sectors of the population. UK-based studies have included relatively few non-white participants, making it difficult to explore ethnic differences. Some of the studies were designed to be population representative, and so this was expected from the outset.^12^ ^13^ It also seems that lower response rates occurred for ethnic minority groups.^3^ ^4^ Only one study has assessed vaccine uptake in the UK, and it found lower levels in schools with a higher proportion of pupils from ethnic minority backgrounds.^14^

It is essential that uptake of the HPV vaccine is equitable, or the immunisation programme could widen ethnic disparities in health. A first step is therefore to assess ethnic differences in acceptability of the vaccine, and to try to uncover the reasons for any differences. We carried out a survey recruiting participants from the major ethnic minority groups in the UK, with the aims of considering ethnic differences in awareness of HPV and acceptability of HPV vaccination.

## Materials and methods

### Participants

Data were collected in two waves of the monthly omnibus survey conducted by Ethnibus (July and August 2008). Ethnibus (http://www.ethnicfocus.com) recruits participants from the largest ethnic minority groups in proportion to their representation in the UK population. Within each ethnic group, quota-based age distributions are fulfilled. The present sample had a total of 750 female participants from six ethnic minority groups (Indian, Pakistani, Bangladeshi, Caribbean, African and Chinese) plus 200 white British women.

Postal areas with high proportions of residents from each ethnic group were randomly selected using 2001 census data for England (mostly in London and the Midlands). Multilingual interviewers visited households in these areas, and individuals, selected based on their self-reported ethnicity, were invited to participate. Data were collected during face-to-face interviews, and each participant received a £5 incentive. Ethnibus reported that approximately 70% of individuals who met the eligibility criteria agreed to be interviewed (based on figures recorded by the interviewers), and this was similar to response rates in other Ethnibus surveys. The research was exempt from formal ethics committee approval because it ensured complete anonymity.

### Measures

All participants were given HPV information based on the Department of Health’s leaflet designed for girls aged 12–13 years and their mothers. This was translated into the most common languages (Urdu, Bangla, Hindi and Mandarin) by agency-recruited qualified translators, translating into their first language. For all the other languages (Punjabi, Gujarati, Somali, Sylethi, Uroba, Lugani, Arabic, French and Portuguese), the information was verbally translated during the interview. Questions were developed from previous research and qualitative interviews with 20 ethnic minority mothers.^15^ They were then piloted with 45 women to ensure clarity. The information provided and questions asked are available online as Supplementary material.

After hearing the information, women were read the following: “If you have a daughter age 12–13 years, please think about her when answering the questions; if you don’t have a daughter age 12–13 years, please imagine that you do. Starting in September 2008, girls age 12–13 years (school year 8) will be offered the HPV vaccination in school. If your daughter were invited to have the HPV vaccination at school this autumn, would you agree to her having it.” Responses were made on a five-point scale (definitely not–yes definitely). Women were asked to give a reason for their answer which was recorded verbatim. All participants were asked to indicate the father’s role in deciding about HPV vaccination because qualitative work indicated possible ethnic differences in this.^15^ At the end of the interview, women were asked: “Before this interview had you heard of HPV (human papillomavirus)”, with response options of yes, no and don’t know. This question was asked at the end of the interview in order to avoid confusion regarding similar terms (eg HIV) and to encourage women who had heard of HPV but did not remember the name to say yes.

Socioeconomic class (SEC) was based on the occupation of the chief earner in the household and was coded: AB managerial/professional; C1 supervisory; C2 skilled manual; D semi-skilled/unskilled manual; E state pensioners or casual/lowest grade workers. These groupings are often used in market research.^16^ Age group, marital status, religion and language spoken at home were also assessed. An additional question asked about generational status, classifying women as “second generation” (UK-born and parents UK-born), “first generation” (UK-born but at least one parent non-UK-born) or “immigrant” (non-UK-born and parents non-UK-born). This question was from the Millennium cohort study, a prospective study of children in the UK.^17^ Generational status is an indicator of acculturation and is an important issue when studying ethnic minorities in the UK. It has been associated with maternal health behaviours (eg breast-feeding), and we expected it to influence the acceptability of HPV vaccination. For mothers who are second generation, both they and their parents were born, brought up and educated in Britain, and they would therefore be expected to have more westernised attitudes. Finally, women indicated whether they had any daughters and what ages they were.

### Analysis

Data were analysed using SPSS 15.0. HPV awareness and HPV vaccine acceptability were recoded into binary variables. Women responding to the HPV awareness question were categorised as “aware of HPV” (yes) or “not aware of HPV” (no/don’t know). Mothers responding to the HPV vaccine acceptability question were coded as “acceptors” (yes probably/yes definitely) or “non-acceptors” (probably not/definitely not/unsure). Initially, mothers who were “unsure” were considered as a separate group but, as they were not distinctly different from those who said probably or definitely not, the two groups were combined. Univariate logistic regression analyses were run to identify predictors of HPV awareness and vaccine acceptability. Significant univariate predictors were entered into a multivariate model.

A coding frame was developed (by LM) and applied (by LM and AF) to the mothers’ verbatim reasons for their decision. Inter-rater reliability was “outstanding” (kappa  = 0.94). χ^2^ analyses were used to explore reasons for the vaccine decision (cited vs not cited) and involvement of the father (mother takes lead, father takes lead, decide together). For each analysis, different ethnic groups were compared with the white group.

## Results

### Sample characteristics

The sample included 950 women: white British (n = 200), Indian (n = 235), Pakistani (n = 164), Bangladeshi (n = 63), Caribbean (n = 130), African (n = 107) and Chinese (n = 51). Sample characteristics are shown in table 1. Most women were aged 25–34 years (26%) or 35–44 years (28%), were married (66%) and were in socioeconomic classes C or D (supervisory, 24%; or manual workers, 49%). Most women reported some religious affiliation (87%), with the majority being Christian (30%) or Muslim (36%). There were roughly equal proportions of “second generation”, “first generation” and “immigrants” among Indian, Caribbean, African and Chinese women. There were fewer second-generation Pakistanis and Bangladeshis (15% and 6% respectively). Most respondents spoke English at home (68%), but this varied by ethnicity and generational status.

**Table 1:** Sample characteristics for women in each ethnic group (column percentages)

	White (n = 200)	Indian (n = 235)	Pakistani (n = 164)	Bangladeshi (n = 63)	Caribbean (n = 130)	African (n = 107)	Chinese (n = 51)
**Age (years)**							
16–24	10	20*	23*	29*	22*	20*	24*
25–34	29	**29**	**24**	**24**	**24**	**30**	**18**
35–44	25	**26**	**34**	**25**	**22**	**35**	43*
45–54	17	**12**	**10**	**16**	**15**	**9**	**16**
55+	21	**15**	10*	6*	**17**	7*	0*
**Marital status**							
Married/cohabitating	73	63*	**71**	**78**	52*	**62**	**71**
Single	21	**25**	**25**	8*	42*	34*	**22**
Divorced/separated/widowed	7	**12**	**4**	**14**	**5**	**5**	8
**Socioeconomic class**							
AB Managerial/professional	15	**18**	7*	**21**	**7**	6*	**8**
C1 Supervisory	18	27*	28*	**21**	**22**	**22**	**28**
C2 Skilled manual	29	**27**	**35**	**30**	**29**	**19**	**31**
D Semi-skilled/unskilled manual	22	**16**	**14**	**18**	**28**	**29**	**14**
E State pensioners/unemployed	17	**12**	**15**	**11**	**15**	**24**	**20**
**Religion**							
Christian	64	9*	0.0*	0*	76*	36*	8*
Hindu	0	56	0	0	0	0	0
Sikh	0	17	0	0	0	0	0
Muslim	0	18	100	100	0	64	0
Other	4	0	0	0	0	0	35
None	33	0	0	0	24	1	53
**Language**							
English	100	57*	51*	67*	100	38*	27.5*
Punjabi	0	17	13	0	0	0	0
Hindi	0	9	0	0	0	0	0
Gujarati	0	13	0	0	0	0	0
Urdu	0	5	36	0	0	0	0
Bangla	0	0	0	22	0	0	0
Mandarin	0	0	0	0	0	0	73
Somali	0	0	0	0	0	49	0
Other	0	0	0	11	0	13	0
**Generational status**							
Second generation	94	26*	15*	6*	35*	24*	28*
First generation	6	32	54	48	29	36	33
Immigrant	0	43	32	46	35	40	39

**Bold** type represents when a 2×2 χ^2^ was used to analyse the difference in that ethnic group compared with the white British group. Where figures are not in bold type, it was not possible to compute χ^2^ results because more than 20% of cells in the analysis had a count of less than 5.

*p<0.05.

### Awareness of HPV

Overall, 17% of women reported (at the end of the interview) that they had heard of HPV before taking part in the study. Univariate logistic regression explored predictors of HPV awareness; odds ratios (ORs) and confidence intervals (CIs) are shown in table 2. There was no association between SEC and awareness of HPV, but there was a slight difference by age, with higher awareness in women aged 16–24 years than in women aged 35–44 years (22% vs 13%). There was also a difference in relation to marital status, with higher awareness in single than in married women (21% vs 14%). There was wide variation across ethnic groups, with 39% of white British women having heard of HPV, but much lower awareness in all ethnic minority groups: Chinese (18%), Caribbean (14%), Pakistani (12%), Indian (9%), African (8%) and Bangladeshi (6%). Religion, generational status and language spoken at home were also predictors of awareness in univariate analyses. Fewer Muslim (10%) and more Christian women (27%) were aware of HPV than women with no religion (18%). There was greater awareness among “second-generation” women (24%) than “first-generation” (14%) or “immigrant” women (10%), and women who spoke English at home were more likely to have heard of HPV (19%) than those who spoke another language (11%). In multivariate logistic regression, age, ethnicity and religion remained significant (overall model: χ^2^(18) = 123.76, p<0.001; Nagelkerke R^2^ = 0.21). Marital status, language and generation were no longer significant.

**Table 2:** Univariate logistic regression analyses exploring predictors of HPV awareness (n = 950) and acceptability of HPV vaccination (mothers only, n = 601)

	Awareness of HPV			Acceptability of HPV vaccination		
	%	OR (95% CI)	p Value	%	OR (95% CI)	p Value
**Ethnicity**						
White British	39	1.00		63	1.00	
Indian	9	0.15 (0.09 to 0.26)	<0.001	25	0.20 (0.12 to 0.33)	<0.001
Pakistani	12	0.22 (0.13 to 0.38)	<0.001	11	0.07 (0.04 to 0.14)	<0.001
Bangladeshi	6	0.11 (0.04 to 0.30)	<0.001	18	0.13 (0.06 to 0.29)	<0.001
Caribbean	14	0.25 (0.14 to 0.45)	<0.001	49	0.55 (0.32 to 0.98)	0.040
African	8	0.13 (0.08 to 0.27)	<0.001	51	0.61 (0.33 to 1.10)	0.102
Chinese	18	0.34 (0.16 to 0.73)	0.006	40	0.39 (0.18 to 0.87)	0.022
**Age (years)**						
16–24	22	1.78 (1.08 to 2.93)	0.024	17	0.38 (0.14 to 1.02)	0.054
25–34	18	1.42 (0.88 to 2.30)	0.149	43	1.43 (0.94 to 2.16)	0.091
35–44	13	1.00		35	1.00	
45–54	19	1.55 (0.88 to 2.74)	0.128	36	1.06 (0.63 to 1.77)	0.834
55+	11	0.81 (0.42 to 1.56)	0.527	43	1.42 (0.87 to 2.32)	0.167
**Marital status**						
Married/cohabitating	14	1.00		37	1.00	
Single	21	1.60 (1.09 to 2.33)	0.015	36	0.95 (0.55 to 1.65)	0.848
Divorced/separated/widowed	23	1.65 (0.91 to 3.00)	0.099	43	1.24 (0.68 to 2.28)	0.480
**Socioeconomic class**						
AB Managerial/professional	21	1.00		38	1.00	
C1 Supervisory	17	0.78 (0.44 to 1.38)	0.400	34	0.81 (0.46 to 1.43)	0.471
C2 Skilled manual workers	16	0.74 (0.43 to 1.29)	0.293	34	0.83 (0.48 to 1.42)	0.486
D Semi-skilled/unskilled manual workers	15	0.67 (0.37 to 1.22)	0.187	38	0.98 (0.55 to 1.73)	0.935
E State pensioners/unemployed	16	0.73 (0.39 to 1.37)	0.325	48	1.48 (0.83 to 2.66)	0.187
**Religion**						
None	18	1.00		64	1.00	
Christian	27	1.73 (1.02 to 2.93)	0.043	53	0.61 (0.37 to 1.03)	0.066
Hindu	12	0.61 (0.30 to 1.24)	0.173	34	0.28 (0.15 to 0.53)	<0.001
Muslim	10	0.51 (0.23 to 0.92)	0.024	18	0.12 (0.07 to 0.21)	<0.001
Other	16	0.93 (0.42 to 2.05)	0.855	17	0.12 (0.04 to 0.33)	<0.001
**Language**						
English	19	1.00		44	1.00	
Not English	11	0.52 (0.35 to 0.79)	0.002	26	0.45 (0.31 to 0.65)	<0.001
**Generational status**						
Second generation	24	1.00		50	1.00	
First generation	14	0.51 (0.34 to 0.76)	0.001	35	0.54 (0.36 to 0.80)	0.002
Immigrant	10	0.37 (0.23 to 0.57)	<0.001	25	0.34 (0.22 to 0.51)	<0.001
**Aware of HPV**						
Yes	–	–	–	41	1.00	
No	–	–	–	37	0.83 (0.52 to 1.32)	0.432

### HPV vaccine acceptability

Just under half the sample (n = 440) had a daughter under 16 years old and, of these, 137 had a daughter aged 12–13 years. An additional 17% (n = 161) had a daughter aged 16 years or older. Acceptability did not differ based on the daughter’s age, so all women with a daughter, regardless of her age, were included in the analysis of acceptability (n = 601). Overall, 38% of mothers were “acceptors” (21% yes definitely; 17% yes probably) and 62% were “non-acceptors” (18% probably not; 19% definitely not; 25% “unsure”). Univariate logistic regression explored predictors of HPV vaccine acceptability (ORs and CIs are shown in table 2). There were no associations between HPV vaccine acceptability and age, marital status or SEC. Acceptability varied by ethnic group, being highest in white women (63%) followed by African, Caribbean and Chinese women (51%, 49% and 40%), and much lower among Indian, Bangladeshi and Pakistani women (25%, 18% and 11%). Acceptability also varied on the basis of generational status and whether English was spoken at home. It was higher in “second-generation” (50%) than “first-generation” migrants (35%) and “immigrants” (25%), and among mothers who spoke English at home (44%) than among those who spoke another language (26%). Religion was associated with acceptability, with fewer “acceptors” among Hindus (34%) and Muslims (18%) compared with those with no religion (64%). In multivariate analyses, which included SEC, only ethnicity and religion remained significant (overall model: χ^2^(13) = 129.14, p<0.001; Nagelkerke R^2^ = 0.26).

### Reasons for intended vaccine decision

Table 3 shows the reasons that mothers cited for their intended vaccine decision. The most common reasons for giving the vaccine were: the protection it would offer from “HPV” or “cervical cancer”, health reasons, for example for their daughter’s “health”, “safety” or “well-being”, and because of a general positive response to the vaccine, for example “I feel positive about it”. Other reasons included “peace of mind”, “because there is a need for it”, “because it will be made freely available” and “because it is part of a parent’s responsibility”.

**Table 3:** Reasons for HPV vaccine decision (percentage of mothers citing each reason, n = 601)

	White (n = 149)	Indian (n = 130)	Pakistani (n = 112)	Bangladeshi (n = 45)	Caribbean (n = 74)	African (n = 61)	Chinese (n = 30)
**Reasons for accepting**							
Protection	29	16*	11*	13*	14*	**25**	**30**
Daughter’s health	9	**6**	**5**	2	**16**	**7**	7
General positive beliefs	5	**2**	**3**	0	7	7	0
Benefits	5	**3**	5	0	3	7	0
Availability	3	1	0	0	3	0	3
There is a necessity	2	1	1	0	1	0	0
Responsibility	3	0	0	0	1	0	0
Peace of mind	0	0	1	0	3	2	0
**Reasons for not accepting**							
More information needed	16	**12**	**21**	44*	**18**	**10**	**20**
Sex-related reasons	2	15*	20*	4	16	5	10
Safety/side-effects	7	**12**	**6**	13	5	5	3
Religion/culture	5	**8**	**6**	0	3	7	3
No necessity	0	8	5	2	0	2	0
Age-related reasons	1	2	1	4	1	3	0
Trust	0	2	5	0	0	2	0
Discuss with family	1	2	1	0	0	2	0

**Bold** type represents when a 2×2 χ^2^ was used to analyse the difference in that ethnic group compared with the white British group. Where figures are not in bold type, it was not possible to compute χ^2^ results because more than 20% of cells in the analysis had a count of less than 5.

* p<0.05.

The most common reasons for declining were: needing more information (either about “side-effects” or generally), sex-related concerns (eg “it encourages promiscuity” or “risk of premature sex”), religious/cultural reasons (eg “we’re very religious, no sex before marriage”) and concerns about safety. Other reasons not to give the vaccination included believing it would be unnecessary or inappropriate, age-related reasons (eg 12–13 years is too young), lack of trust in vaccinations and wanting to discuss the decision with other family members.

We explored whether some ethnic groups were more or less likely to cite specific reasons for their decision. Citing the “protection” that the vaccine would offer occurred less among Indian (16%), Pakistani (11%), Bangladeshi (13%) and Caribbean mothers (14%) compared with white mothers (29%), but there were no differences between white and African or Chinese mothers. Needing more information was cited more by Bangladeshi mothers than by white mothers (44% compared with 16%), but there were no differences between white mothers and mothers from any other backgrounds. We were able to look at differences for Indian and Pakistani mothers in citing sex-related reasons, religious reasons, daughter’s health and future benefits. Sex-related reasons were more likely to be cited by both Indian (15%) and Pakistani mothers (20%) than white British mothers (2%). None of the other reasons differed significantly. Limited numbers meant that differences could not be explored for Bangladeshi, Caribbean, African and Chinese women (more than 20% of cells in the χ^2^ analyses had an expected count of less than 5).

### The role of fathers in deciding about HPV vaccination

Table 4 shows the mothers’ responses to the question about fathers’ involvement in the decision to vaccinate against HPV. Overall, 41% of mothers said they would take the lead in deciding about HPV vaccination, and 52% said they would make a joint decision with the father. Again, this varied by ethnic group, with a shared decision most likely in Indian (61%), Pakistani (57%), Bangladeshi (66%), African (63%) and Caribbean groups (56%) and least likely in Chinese (30%) and white groups (36%). In comparison with white British mothers, all ethnic minorities except the Chinese were more likely to share the decision. A small proportion (7%) said the father would take the lead role in deciding about HPV vaccination, and this was slightly more common among Chinese (17%), Bangladeshi (16%), Pakistani (10%) and Indian (9%), followed by African (5%) and Caribbean mothers (3%). None of the white mothers said the father would take the lead role in the decision (χ^2^ results are not reported for this finding because more than 20% of cells in the analysis had a count of less than 5).

**Table 4:** Fathers’ roles (column percentages)

	White (n = 149)	Indian (n = 130)	Pakistani (n = 112)	Bangladeshi (n = 45)	Caribbean (n = 74)	African (n = 61)	Chinese (n = 30)
I would take the lead role	64	31*	32*	18*	42*	32*	**53**
We would decide together (50:50)	36	61*	57*	66*	56*	63*	**30**
Her father would take the lead role	0	9	10	16	3	5	17

**Bold** type represents when a 2×2 χ^2^ was used to analyse whether the reason was cited more/less in that ethnic group compared with the white British group.

*Indicates a significant difference, p<0.05.

## Discussion

Acceptability of HPV vaccination for a 12- to 13-year-old daughter was much lower in ethnic minority mothers than in white mothers, with the lowest levels among mothers from South Asian backgrounds. This was the case even after controlling for SEC. This suggests that previous research with predominantly white British parents cannot be extrapolated to ethnic minority parents, emphasising the importance of including minority women in future research. If these findings map on to uptake, it may mean that some areas of the UK achieve particularly low uptake. The non-white population accounts for 8% across Britain, but this rises to 30% in some areas (Slough, Leicester, Luton, Birmingham and London), and is even greater in parts of London (eg Brent, 71%; Newham, 66%).^18^ To date, collection of ethnicity data by the NHS has been limited, but this is expected to improve,^19^ and the parental consent form for HPV vaccination in England includes a box to record ethnicity. These findings support the importance of monitoring ethnicity in relation to uptake of the HPV vaccination.

Generation and language spoken at home were not significant predictors of acceptability in multivariate analyses, suggesting that acculturation does not mediate the effect of ethnicity. However, religion remained an important factor, and “religious reasons” were one of the main reasons cited for declining the vaccine. This is consistent with previous work that found parents with “strong religious or cultural views” were less likely to accept HPV vaccination.^3^ It is also consistent with attitudes to HPV testing, which some minority women felt reflected “non-traditional cultural or religious practices concerning sex and monogamy”.^20^ The importance of religion appears to come from a strong belief in sexual abstinence until marriage, and this is a barrier that will be a challenge to overcome. There is evidence that South Asian women have fewer sexual partners than white women; nonetheless, 13–29% report more than one lifetime sexual partner, and this is nearly 50% in South Asian men.^21^ Liaising with religious groups about the best ways to communicate HPV information may help to make HPV vaccination more acceptable.

Across the board, one of the most common barriers to accepting HPV vaccination was concern about side-effects. This is consistent with a recent study of HPV vaccine uptake,^14^ and was important to all mothers regardless of ethnicity. An additional reason given “against” HPV vaccination was the belief that the vaccine would “lead to early sex” or “encourage promiscuity”, which again has been suggested as a reason for non-acceptability in numerous other studies.^3^ ^4^ ^6^ ^8^ ^10^ ^11^ This concern was cited more by South Asian women.

We decided to ask about awareness of HPV at the end of the interview, so that women knew what HPV was before saying whether they had heard of it. This makes it unlikely that levels of awareness are underestimated in this study, and means that the findings may not be comparable to other studies which have assessed it at the beginning of the interview. Ethnicity was associated with HPV awareness, with much lower levels in all the ethnic minority women, particularly Indian, African and Bangladeshi women (<10%). Interestingly, language spoken at home did not explain this effect, suggesting that it is not purely the result of a language barrier. One alternative explanation could be variation in exposure to publicity about HPV because of lower coverage in culturally specific media or because sex-related topics are considered “taboo” and as a consequence are discussed less in some cultures. Given that knowledge of HPV is necessary for informed decision-making about vaccination, tailoring information campaigns and channelling these through media outlets aimed at specific ethnic groups may help to raise awareness.

One additional finding of interest was that ethnic minority mothers were more likely to believe the father would play an important role in the decision to vaccinate against HPV. To date, there has been little research into fathers’ attitudes in the UK, and the Department of Health leaflet about HPV has been aimed at girls and their mothers. Future research exploring parental decision-making with regard to HPV vaccination among ethnic minorities should include fathers.

There are several limitations to this study. The sampling method means the findings may not be generalisable to all ethnic minority mothers in the population, although the quota sampling technique allowed us to focus on and compare ethnic minority groups. The response rate of 70% was good; however, the figure is based on details recorded by the field interviewers. As the interviewers sometimes forget to report these response details, Ethnibus describe the response rate as “approximate” rather than exact. The response rate should therefore be interpreted with caution. Another issue is that, although all mothers in the acceptability analysis had a daughter, not all daughters were aged 12–13 years, and this meant that some mothers were responding hypothetically. Third, we did not assess actual vaccine uptake, and statements about intention can only be indicative of acceptance of HPV vaccination. Expected vaccine acceptance in this sample of white mothers was 63%, which was lower than the 75% and 81% that have been found in other studies.^3^ ^4^ However, in previous surveys, parents were asked to return a questionnaire about vaccination, and thus these studies may have received responses only from the most “motivated” parents, whereas in the present study, the questions formed part of a larger survey on a range of issues, so attitudes to this topic are unlikely to have biased participation.

Over the coming years, it will be possible to incorporate behavioural outcomes into work on HPV attitudes, but for now exploring acceptability offers some insight into a decision that is facing parents of adolescent girls right now. It gives a head start in understanding possible inequalities before uptake data from the first cohorts of girls are available. Understanding ethnic differences in acceptability of HPV vaccination will be particularly useful to health promotion specialists working in ethnically dense areas of the country, helping them to limit inequalities and support informed choice by all.

**What is already known on this subject**

- HPV vaccination is now offered to all girls aged 12–13 years.
- In one study, uptake was lower in schools with a higher proportion of ethnic minority pupils.
- One criticism of the UK-based studies is their limited inclusion of non-white participants.

**What this study adds**

- Ethnicity and religion are predictors of HPV awareness and acceptability of HPV vaccination.
- Sex-related concerns are more likely to be a barrier among ethnic minority parents.
- The ethnic differences found in this study support the importance of monitoring ethnicity in vaccine uptake data.

## Supplementary Material

Web supplement
