# Supplementary material for: Ethnic differences in human papillomavirus awareness and vaccine acceptability
Source: J Epidemiol Community Health. 2009 Sep 17;63(12):1010–5. doi: 10.1136/jech.2008.085886 (PMC3960938; doi:10.1136/jech.2008.085886)
Supplement: Web supplement [file HZT-63-12-1010-s1.pdf]

**Supplementary online material: Information presented and questions asked to women**

This information is about the new vaccination (injection) that girls can have to protect themselves against cervical cancer when they get older.

Cervical cancer occurs in the cervix (the entrance to the womb). It is caused by a virus called the human papillomavirus or HPV. It can be very serious and around 1000 women die from cervical cancer in the UK each year.

The virus is very common and is caught by being sexually active with another person who has the virus. Because it is so common, most people will get infected at some point in their lifetime. Most of the time, the virus does not cause cancer because it is killed off by the body's immune system, but not always.

A vaccine protecting against the HPV virus is now available, and girls need to have the vaccination before they start being sexually active. While most girls don't start having sex before they're at least 16 or quite a bit older, it is recommended that girls have the vaccine at 12 to 13 years of age, to give them as much protection as possible. The side effects of the vaccination are quite mild – usually just soreness in the arm that soon wears off.

The next few questions are about deciding to give HPV vaccination to girls. If you have a daughter age 12-13 years please think about her when answering the questions, if you don't have a daughter age 12-13 years, please imagine that you do. Starting in September 2008, girls age 12-13 years (year 8) will be offered the HPV vaccination in school.

If your daughter were invited to have the HPV vaccination at school this autumn, would you agree to her having it?

- Definitely not
- Probably not
- Not sure
- Yes, probably
- Yes, definitely

Can you say why you would [definitely/probably/not sure you would/probably not/definitely not] take up the offer? (Recorded verbatim)

We would like to know about fathers' roles in deciding about HPV vaccination for their daughters. Please select which statement most applies to you:

- Her father would take the lead role in deciding about this vaccine
- I would take the lead role in deciding about this vaccine
- Her father and I would make the decision about this vaccine together (50:50)
- Not applicable (e.g. father not around)
- Other *(verbatim)*

Before this interview had you heard of HPV (Human Papillomavirus)?

- Yes
- No
- Don't know
